# Integrating Bioinformatics and Machine Learning for Genomic Prediction in Chickens

**DOI:** 10.3390/genes15060690

**Published:** 2024-05-26

**Authors:** Xiaochang Li, Xiaoman Chen, Qiulian Wang, Ning Yang, Congjiao Sun

**Affiliations:** State Key Laboratory of Animal Biotech Breeding and Frontiers Science Center for Molecular Design Breeding (MOE), China Agricultural University, Beijing 100193, China; lixc1219@126.com (X.L.); xxmchen@outlook.com (X.C.); s20233040766@cau.edu.cn (Q.W.); nyang@cau.edu.cn (N.Y.)

**Keywords:** genomic prediction, machine learning, GWAS, poultry breeding

## Abstract

Genomic prediction plays an increasingly important role in modern animal breeding, with predictive accuracy being a crucial aspect. The classical linear mixed model is gradually unable to accommodate the growing number of target traits and the increasingly intricate genetic regulatory patterns. Hence, novel approaches are necessary for future genomic prediction. In this study, we used an illumina 50K SNP chip to genotype 4190 egg-type female Rhode Island Red chickens. Machine learning (ML) and classical bioinformatics methods were integrated to fit genotypes with 10 economic traits in chickens. We evaluated the effectiveness of ML methods using Pearson correlation coefficients and the RMSE between predicted and actual phenotypic values and compared them with rrBLUP and BayesA. Our results indicated that ML algorithms exhibit significantly superior performance to rrBLUP and BayesA in predicting body weight and eggshell strength traits. Conversely, rrBLUP and BayesA demonstrated 2–58% higher predictive accuracy in predicting egg numbers. Additionally, the incorporation of suggestively significant SNPs obtained through the GWAS into the ML models resulted in an increase in the predictive accuracy of 0.1–27% across nearly all traits. These findings suggest the potential of combining classical bioinformatics methods with ML techniques to improve genomic prediction in the future.

## 1. Introduction

Chickens (Gallus gallus) are a crucial source of food for humans and are currently the most economically significant animals in the poultry industry. Breeders aim to enhance the production of chicken eggs and meat to meet the increasing demand for high-quality and cost-effective proteins. Over the past two decades, scientists have progressively integrated genomic data into the best linear unbiased prediction (BLUP) model from genomic selection, as well as refined and optimized BLUP models for diverse application scenarios [1,2,3,4]. The intricacy of animal genomes presents challenges in handling genomic high-throughput data (e.g., single nucleotide polymorphisms, SNPs), in contrast to the relative simplicity and controllability of plant genomes [5]. Classical linear mixed models face challenges in accommodating the complex relationships present in large, noisy, and redundant animal genomic data while tackling the lack of adequate observational data and predictors [6,7]. Additionally, focusing solely on additive effects does not fulfill the requirements for accurate phenotype prediction. Hence, there is a continual necessity to explore more suitable and precise genomic selection and prediction algorithms.

Many researchers have recently proposed genomic selection and prediction models based on machine learning (ML) algorithms that are suitable for various breeding scenarios [8,9]. In studies of pigs, cattle, and broilers, researchers performed genomic predictions on different economic traits through various ML methods [10,11,12,13]. However, a common issue is that the feature engineering and parameter selection process almost completely ignores classical bioinformatics methods, resulting in the poor interpretability of these models, particularly those based on neural networks [14,15]. Additionally, animal genomic data often comprises over 10K SNPs, leading to excessive model parameter fitting in both ML and deep learning models, causing dimension explosion and gradient disappearance [16]. Therefore, prior to utilizing ML algorithms for genomic selection, classical bioinformatics methods can be employed to screen SNPs, thereby enhancing the reliability and interpretability of ML models.

The primary objective of this study was to investigate the suitability and reliability of different ML methods for developing genomic prediction models for various economic traits in chickens. Additionally, we aimed to assess the impact of SNP sets selected using distinct bioinformatics methods on the ultimate fitting outcomes. This study is anticipated to generate novel insights and perspectives for genomic prediction in animal breeding, thus assuming a pivotal role in chicken breeding.

## 2. Materials and Methods

### 2.1. Data Collection

The sample dataset was obtained from the Rhode Island Red pure line population of Beijing Huadu Yukou Poultry Industry Co., Ltd. (Beijing, China), and they provided the phenotype statistics and pedigree-based heritability. This line has undergone more than 10 generations of high-intensity artificial selection for egg production traits. We selected 4190 female chickens with phenotype records of 10 traits, including body weight (BW), egg number (EN), egg weight (EW), eggshell color (ESCA, ESCB, ESCI, and ESCL), eggshell strength (ESS), albumen height (AH), and egg glossiness (SINS) at different ages. Blood samples of all chickens were collected from the wing and placed in an anticoagulant tube containing EDTA. The integrity of the DNA was verified, and the genotypes were captured using an illumina 50K SNP chip (Phoenix No. 1) [17]. The chip was established based on the reference genome of Gallus_gallus-5.0, and all loci were converted to Gallus_gallus-6.0 by Liftover (https://genome.ucsc.edu/cgi-bin/hgLiftOver, accessed on 15 February 2024) [18]. All animal experiments were approved by the Animal Care and Use Committee of China Agricultural University (permit number SYXK 2007-0023). All animal experiments were performed strictly according to the requirements of the Animal Ethics Procedures and Guidelines of the People’s Republic of China.

### 2.2. Quality Control and Imputation

We used PLINK v1.90 [19] to perform quality control on genotype data, including MAF, SNP call rate, and individual call rate. The criteria for all filtering methods were set to 0.05 (--maf 0.05 --geno 0.05 --mind 0.05), and we ultimately obtained 36,985 high-quality SNPs to participate in subsequent imputation via BEAGLE v5.2 [20].

### 2.3. Genome-Wide Association Study (GWAS)

We conducted genome-wide association studies (GWASs) on all traits, incorporating all valid samples and SNPs, using a univariate linear mixed model (LMM) implemented in GCTA v0.98.6 software [21]. To address population stratification, we employed the genomic relationship matrix (GRM) and considered the first five dimensions of principal components (PCs) as covariates. The statistical model employed in this investigation is presented by the following equation:

**y** = **Wα** + **xβ** + **μ** + **ε**, (1)

Here, **y** represents the phenotype records of 4190 individuals. **W** is a matrix of covariates (fixed effects: top five principal components), accounting for population structure, with **α** being a vector of corresponding effects that form the intercept; **x** signifies the marker genotypes; **β** refers to the associated marker effects; **μ** constitutes a vector of random polygenic effects with a covariance structure; and **ε** denotes a vector of random residuals. The *p* value served as a benchmark to assess the significance of the association between SNPs and the traits. The threshold for genome-wide significance was defined using a modified Bonferroni correction, applied using the R package simpleM [22]. A total of 1509 independent SNPs were examined, and the thresholds for genome-wide significance and suggestive significance were 3.31 × 10^−5^ (0.05/1509) and 6.63 × 10^−4^ (1/1509), respectively.

### 2.4. Functional Annotation

We employed the online website g:Profiler (https://biit.cs.ut.ee/gprofiler/gost, accessed on 5 March 2024) [23] to retrieve enriched functional terms for these genes, including Gene Ontology (GO) categories and KEGG pathways.

### 2.5. Feature Engineering

In the current study, we used each of the three methods provided by PLINK for feature engineering. We utilized --pca *n* (*n* = 10, 30, 50, 100, 200, and 500) to achieve different degrees of parameter dimension reduction. We employed --maf *x* (*x* = 0.1, 0.2, 0.3, 0.4) to derive a parameter set with different minimum allele frequencies. We utilized --indep-pairwise *25 5 r*^2^ (*r*^2^ = 0.1, 0.2, 0.3, 0.4, 0.5) to identify various levels of linkage disequilibrium SNPs and obtained the independent loci.

In addition to the three methods described above, we composed the significant and suggestive significant loci for each trait obtained from the GWAS into a subset of SNPs for the corresponding trait, which were used to fit the trait.

### 2.6. Model Building

Following genotyping and feature engineering, we used an automatic ML software package, AutoGluon v1.0.0 [24], to construct multi common ML algorithms, including Random Forest (RF), Extra Tree (ET), K-nearest neighbors (KNNs), CatBoost (CAT), LightGBM (LGB), and neural networks (NNs), to fit different sets of SNPs and model 10 economic traits of chickens for comparison with the accuracy and bias of prediction of the ridge regression BLUP (rrBLUP) [25] and BayesA methods [26]. All models reduced overfitting by 10-fold cross-validation.

The rrBLUP models for genomic prediction were developed using the R package “rrBLUP” (version 4.6.2). This model employs ridge regression methods to estimate marker effects and effectively addresses potential multicollinearity issues in the genetic correlation matrices. We first converted the genotype file from the bed format to the raw format using the --recodeA command in PLINK. The raw format files were imported into R and divided into train and test sets. We then fitted input files using the mixed.solve() function in rrBLUP. After obtaining the predicted values, the Pearson correlation coefficient and the root-mean-square error (RMSE) between the predicted values and the raw phenotypes were calculated using the cor() function and the rmse() function of the “Metrics” package, respectively.

The BayesA models were developed using the R package “BGLR” (version 1.1.2). After importing the genotype and phenotype into R, we employed the BGLR() function to fit the input files with the parameters set as nIter = 50,000, burnIn = 5000, and thin = 10, which specified the total number of iterations for the MCMC sampling, the number of iterations to discard at the beginning of the MCMC sampling process, and the interval for sampling, respectively.

We used the open-source AutoML toolkit AutoGluon v.1.0.0 to construct genomic prediction models based on ML algorithms. ML models utilized in this study encompassed RF, ET, CAT, KNN, LGB, NN, and stacking structures derived from these algorithms by linear fitting and were named Weighted Ensemble (WE). All of these models have been shown in some animal or plant datasets to potentially improve the accuracy of genomic prediction [10,27,28,29]. The toolkit’s hyperparameter optimization function automatically fine-tuned model parameters to select the best-performing model by the order TabularPredictor().fit(presets = ‘best_quality’). Therefore, we chose the Pearson correlation coefficient and the RMSE as the evaluation metrics for validation data by setting eval_metric = pearsonr or root_mean_squared_error and the default hyperparameters for each ML model mentioned above (Table 1). The 10-fold cross-validation was carried by the order num-bag-folds = 10.

## 3. Results

### 3.1. Phenotype Statistics and Genome-Wide Association Study (GWAS)

**Figure 1:**
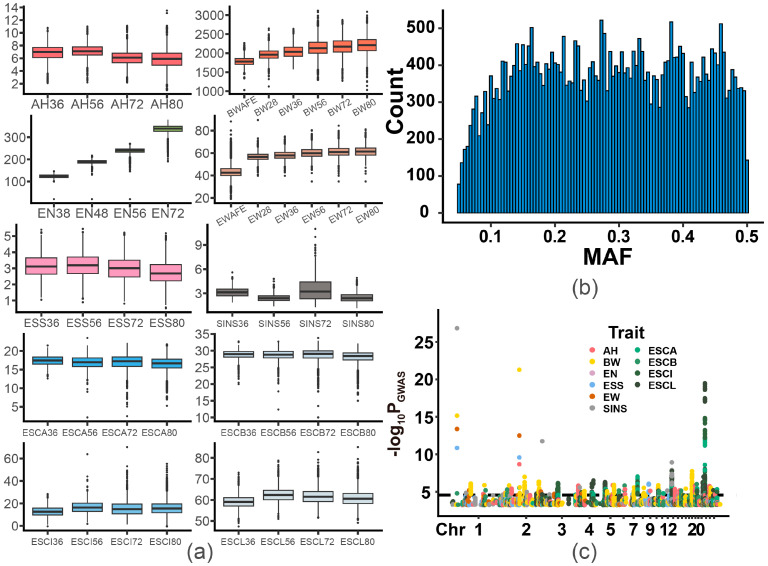
Phenotype statistics and genome-wide association studies. (**a**) Box plots of all the candidate traits. (**b**). Histogram of the MAF of all SNPs passing the quality control. (**c**). Manhattan plots for associations of suggestive significant SNPs with all candidate traits. The threshold for genome-wide suggestive significance was 3.18 (*p* value = 6.63 × 10^−4^). The horizontal black line indicates the genome-wide significance thresholds (*p* values = 3.31 × 10^−5^, threshold = 4.48). Different colors represent different traits.

Phenotype data were collected from a total of 4190 hens for 10 traits at multiple age points, primarily including body weight (BW), egg number (EN), egg weight (EW), eggshell color (ESCA, ESCB, ESCI, and ESCL), eggshell strength (ESS), albumen height (AH), and egg glossiness (SINS). Descriptive statistical analysis revealed the number of reliable samples and the mean, variation, range, and pedigree-based heritability of each trait at different age points (Table 2). Our analysis of phenotype records indicated that the mean BW of the population increased from 1.7 to 2.2 kg with hen aging, and EW increased from 43 to 62 g. For the egg quality-related traits, AH and ESS, showed an increasing and then decreasing trend throughout the laying period (Figure 1a). In addition, ESC (A, B, L, I) and SINS did not show a clear pattern of change. Furthermore, the coefficients of variation (CVs) for most traits increased with age. Notably, the CV values for ESC, SINS, AH, and ESS were the highest among the traits, indicating instability in ESC formation and egg quality in this population. In addition, AH was a low heritability trait (0.157–0.258), and the rest of the traits had medium to high heritability at most time points.

For the genetic analysis, we set the quality control criterion as an MAF greater than 0.05, and the MAF values of all filtered SNPs were evenly distributed between 0.1 and 0.5 (Figure 1b). Following quality control, a GWAS was conducted for each trait, and in total, we identified 953 significant and 3133 suggestively significant SNPs (Figure 1c, Table S1). Significant SNPs associated with BW were concentrated on GGA1, 2, 4, 5, 7, 9, 17, and 24, those associated with EN and EW were concentrated on GGA2, ESS on GGA8, and AH on GGA3, 12, 13, 18, and 24, and those associated with ESC and SINS were more widely distributed on the whole genome. These SNPs were mapped to 1457 genes (Table S2) and 26 reported QTLs corresponding to traits (Table S1). Specifically, 190 genes were associated with BW, 61 with EN, 249 with EW, 421 with ESC, 111 with AH, 98 with ESS, and 78 with SINS. Functional enrichment analysis of the genes related to each trait revealed pathways that correlated well with the observed traits, highlighting a link between these loci and traits (Table 3). Most of the pathways were associated with the regulation of gene expression, intercellular interactions, signal transduction, metabolic regulation, and growth and development. Specifically, the genes related to BW were enriched by the regulation of transcription and expression (*RNA polymerase II transcription regulatory region sequence-specific DNA binding*), mineral uptake and metabolism (*inorganic cation transmembrane transport*), and cellular uptake and metabolism of substances (*regulation of receptor-mediated endocytosis*). Genes related to EN were enriched in intracellular homeostasis (*cellular response to salt*) and histone modification (*MOZ/MORF histone acetyltransferase complex*). Genes related to EW were enriched in affecting cell growth and differentiation (*frizzled binding*), the regulation of metabolic activity (*response to oxygen-containing compounds*), the post-translational modification of proteins (*peptidyl–threonine modification*), and glucose metabolism and lipid synthesis (*cellular response to insulin stimulus*). Genes related to ESS were enriched in calcium channel activity (*voltage-gated calcium channel activity involved in cardiac muscle cell action potential*) and signal transduction (*membrane depolarization during cardiac muscle cell action potential* and *cell–cell signaling involved in cardiac conduction*). 

### 3.2. Machine Learning Methods for Chicken Genomic Prediction

We utilized AutoGluon to establish individual ML models for each trait using the genotype information from all 36,985 loci. Subsequently, we employed rrBLUP and BayesA as references to assess the Pearson correlation coefficients and the RMSE between the predicted values from each method and raw phenotypes. Our preliminary results revealed that when using all loci, the predictive accuracy of all ML methods was notably low, but the prediction biases were relatively high (Figures S1 and S2). Consequently, we conducted PCA to reduce the dimensionality of the genotype data and used the first 100 eigenvalues of the samples as parameters to fit each trait with ML methods. In addition, rrBLUP and BayesA methods performed better when using all loci in predictive accuracy compared to using eigenvalues from PCA, with no significant difference in prediction biases. Therefore, in the latter section of the current study, our focus was on the comparison between the rrBLUP and BayesA methods, which used all loci, and ML methods, which used eigenvalues.

**Figure 2:**
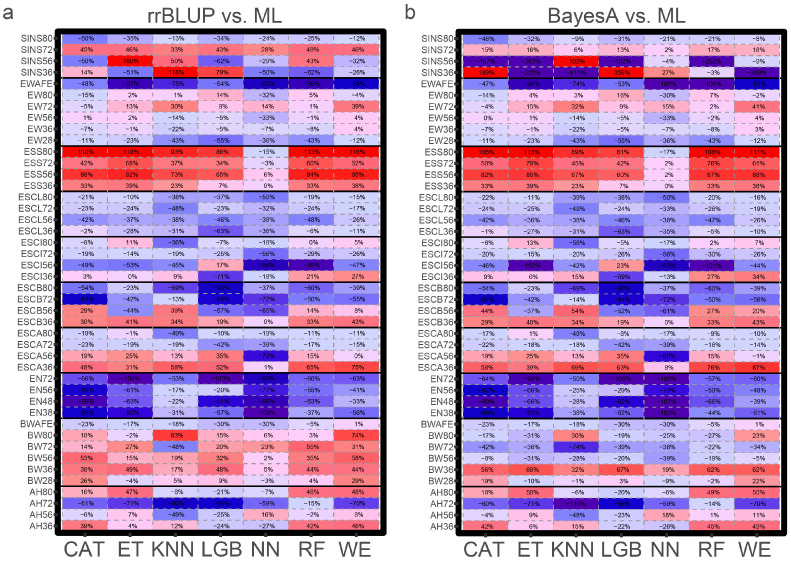
Accuracy of genomic prediction of candidate traits. Comparison of the predictive accuracy of ML algorithms and rrBLUP (**a**) and BayesA (**b**). Red and positive values indicate that the model has an advantage over rrBLUP or BayesA; blue and negative values indicate that rrBLUP or BayesA has an advantage over the methods.

Overall, in all 10 traits and 44 phenotype records, the ML model outperformed rrBLUP and BayesA in 25 phenotypes in predictive accuracy, including all-period ESS, and BW, AH, ESC (A, B, I), EW, and SINS for partial periods (Figure 2). However, the ML model performed significantly worse than rrBLUP and BayesA for all periods of EN and ESCL. Specifically, with the exception of the NN algorithm based on neural networks, the remaining ML algorithms showed a 7~180% higher predictive accuracy in the ESS model compared to the other two methods. In contrast, the predictive accuracies of rrBLUP and BayesA for EN were 17~108% higher than that of the ML algorithms. Among the various ML algorithms, the WE model performed the best in sixteen phenotypes and outperformed rrBLUP and BayesA in twenty phenotypes, and CAT and KNN performed the best in nine and eight phenotypes, respectively (Table 4). 

However, the results of prediction biases, on the other hand, varied considerably. In all phenotypes, the ML model outperformed rrBLUP and BayesA in 26 phenotypes in the RMSE, including all-period BW, EN, ESCB and ESCL, and EW for partial periods, while they performed worse for AH and ESS. Among all ML algorithms, the WE model performed the best in 17 phenotypes and outperformed rrBLUP and BayesA in 25 phenotypes. It is worth noting that the WE model performed best both in predictive accuracy and biases for the pre-body weight (BW28) and mid-term egg weight (EW36, EW56, and EW72).

**Figure 3:**
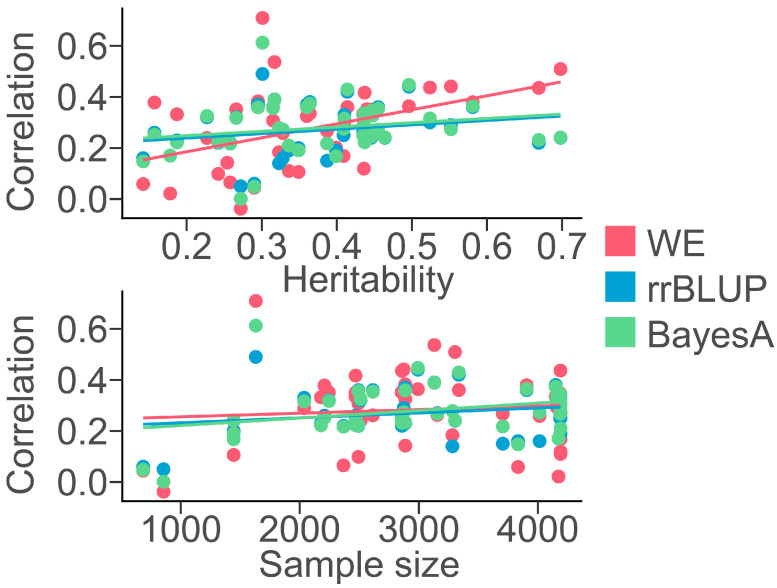
Correlation of heritability and sample size with the predictive accuracy of WE, rrBLUP, and BayesA. Scatter plots of heritability and sample size versus the predictive accuracy of 44 phenotypes.

In addition, we investigated the association between sample size, heritability, and the predictive accuracy of the WE, rrBLUP, and BayesA models (Figure 3). Our findings revealed an absence of a distinct relationship between the sample size and predictive accuracy of all models. Relatively, there was a more pronounced linear relationship between the predictive accuracy and heritability obtained from the WE model compared to rrBLUP and BayesA. This finding was consistent with our expectations, indicating that ML models could capture genetic information for various traits.

### 3.3. Comparison of Different Feature Engineering Methods

In order to investigate the impact of commonly used methods of locus screening on the predictive accuracy of models, we set the gradients on PCA, MAF, and LD pruning to obtain different subsets of SNPs and used these subsets to fit each trait. First, we manipulated the number of principal components (PCs = 10, 50, 100, 200, 300, 400, and 500), and found a consistent pattern where the predictive accuracy of most trait models demonstrated an initial rise followed by a decline as the number of PCs increased, and each trait exhibited distinct peaks at varying PC values (Figure S3). Furthermore, we also applied gradients for MAF (0.1, 0.2, 0.3, 0.4) and LD pruning (*r*^2^ = 0.1, 0.2, 0.3, 0.4, 0.5). In general, the models for most traits did not respond significantly to changes in MAF or LD pruning (Figure S4).

**Figure 4:**
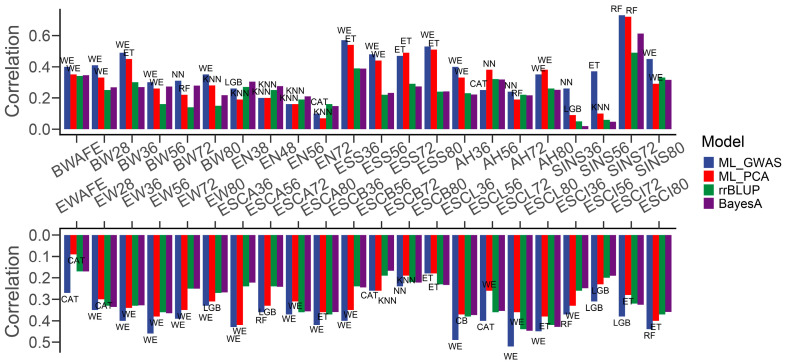
Bar plot of differences in predictive accuracy between suggestive significant SNPs obtained from the GWAS and eigenvalues from PCA. The text above the bars indicates the ML model that obtained the best predictive accuracy.

**Figure 5:**
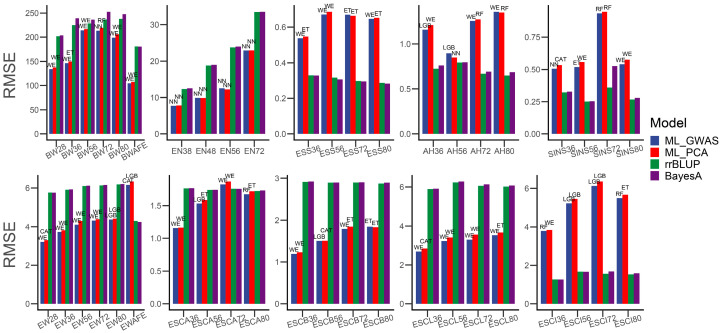
Bar plot of differences in prediction biases between suggestive significant SNPs obtained from the GWAS and eigenvalues from PCA. The text above the bars indicates the ML model that obtained the best prediction bias.

We also utilized the genotypes of suggestively significant SNPs obtained from the GWAS to fit the traits. Interestingly, our analysis revealed that directly using the genotype of these suggestive significant SNPs could lead to an increase in predictive accuracy for almost all phenotypes compared to using the 100 eigenvalues from PCA (Figure 4). Specifically, the predictive accuracy based on the ML methods improved for all but five phenotypes (AH56, AH80, EN48, EN56, and ESS72), with enhancements ranging from 0.69% to 277%. Twenty-nine phenotypes achieved more than 10% improvement, and eighteen achieved more than 20% improvement. In the prediction of pre-intermediate SINS (SINS36, SINS56), training the model with suggestive significant SNPs identified by the GWAS improved the predictive accuracy by 188% and 277%, respectively. In addition, twenty-one phenotypes yielded the same optimal ML model during training on the two parameter sets, with thirteen phenotypes achieving more than 10% improvement. With the exception of the all-period EN, AH56, and ESCB80, the predictive accuracies of the ML methods surpassed those of rrBLUP and BayesA (2~466%), including ESCL, which has not been performed well before. Notably, the WE model sustained its superior performance and remained at the forefront among the 23 phenotypes, particularly in predicting BW, EW, and ESS. On the other hand, there were similar decreases in prediction biases with the ML methods (Figure 5), excluding five phenotypes (EN48, EN56, ESS72, AH80, and ESCB80). But, these decreases were not significant and did not produce qualitative changes for the comparison with the rrBLUP and BayesA methods.

## 4. Discussion

Our research initially highlighted the performance of ten traits in the Rhode Island Red chicken population across multiple time points. As age increased, both BW and EW showed a gradual upward trend; AH and ESS, indicators of egg quality and shell quality, respectively, demonstrated patterns of increase and then decrease. Genetic heritability for all traits, except AH, which was low, ranged from medium to high, aligned with our expectations and previous research findings [30,31,32,33].

Through the GWAS, we identified thousands of significant or suggestive significant SNPs associated with these traits, along with the genes and reported QTLs in which these loci were located. We found that some significant SNPs associated with traits such as BW, EN, AH, and ESC are located in the corresponding reported QTLs. Functional enrichment analyses revealed the biological pathways involved these genes. We found that these genes were commonly associated with pathways, such as the regulation of gene expression, signal transduction, calcium channels, and growth and development, displaying a good correlation between pathways and traits [34,35,36,37]. Both results of the QTL and functional enrichment analyses demonstrated the potential association of the SNPs and genes we found with these traits, providing a foundation for subsequent feature engineering.

This study focused on comparing the accuracy and bias of different genomic prediction methods. Initially employing the genotype of all SNPs to predict phenotypes, we observed that nearly all ML methods yielded poor predictive accuracy and prediction biases. It was hypothesized that this was due to the excessive number of parameters leading to dimensionality explosion, as well as interference by redundant information during model training [38,39]. Consequently, the latter part of this study focuses on how to effectively filter and preprocess SNPs with classical bioinformatics methods to enhance the predictive accuracy and reduce the prediction biases of the ML models.

We attempted to reduce the dimensionality through PCA. PCA is a widely used and efficient dimensionality reduction method in the fields of bioinformatics and machine learning. After training the ML model using the top 100 PCs from the samples, we observed a significant improvement in the results. Of the 44 phenotypes, the ML models outperformed rrBLUP and BayesA more than half of the phenotypes in predictive accuracy, particularly for BW and ESS. However, the models performed poorly in fitting EN and ESCL. Interestingly, the performance of the ML models on the prediction bias of EN and ESS is completely switched around. In predicting EN, the prediction biases of the ML models were significantly smaller than rrBLUP and BayesA, while ESS obtained exactly the opposite result. Most machine learning models outperform linear models in feature extraction and processing. Therefore, the direct reason for the difference in the predictive accuracy of traits is that after dimensionality reduction, the PCs in the population’s genomic information were more strongly related to ESS and showed no significant correlation with EN, making it difficult for the ML models to extract information relevant to EN from the parameters. This might imply that the genetic structure of EN was relatively more linear, while the genetic structure of ESS contained more nonlinear relationships, which contributed to the difference in the performance of ML in the two traits. Since EN is controlled by multiple loci with small effects, even after aggregating the PCs, it may still be impossible to capture the influencing factors related to EN [34,40,41]. On the other hand, the ML models were able to capture the key eigenvalues affecting BW and ESS more effectively. Another possible explanation is that when predicting ESS, ML models were overly sensitive to the noise in the training data, resulting in higher accuracy of its predictions but increased bias in the predicted values, which manifested as overfitting. In contrast, the genetic structure of EN contained less noise, which, in turn, led to better predictive accuracy in linear models. ML models might not have a significant advantage when dealing with cleaner data, but they were able to reduce prediction bias by learning patterns.

Among the ML models used, the WE model performed best. The WE model combined the prediction results from other methods employed in AutoGluon and used linear fitting to generate new predicted values. This modeling framework, known as stacking in ML, places the predictions of multiple first-level models as parameters into a second-level model for re-fitting, making it a widely employed approach for enhancing model predictive accuracy. The CAT and KNN demonstrated good performances. CatBoost is an ML algorithm that excels in processing categorical data, employing a gradient boosting approach, making it well suited for the characteristics of SNP genotype data [42,43,44]. The KNN, on the other hand, is an algorithm based on the principle that “similar entities share attributes” [45]. Considering that researchers of genomic selection and prediction often assume that the same genotypes will have the same effects in a population [1,10,46], the KNN algorithm fits this application scenario well. This could explain its relative success with certain traits, particularly with regard to EN. Compared with other ML algorithms, the KNN has a more obvious advantage in predicting EN. In summary, there is no one model that is superior to others in terms of predictive accuracy across all traits, and there does not seem to be a clear pattern to the variations in predictive accuracy among methods. In linear models, different assumptions about marker effects and their variances are made, and models whose assumptions are closer to the true genetic structure of a particular trait often have higher predictive accuracy [47]. Although ML does not require distributional assumptions on the variables chosen, this situation is also found in ML models, where the predictive performance largely depends on the congruence between model assumptions and the true structure of the data [48]. Therefore, examining the genetic structure of traits through the predictive accuracy of ML models may become a viable method in the future.

Subsequently, we examined the impact of several locus filtering methods on the accuracy of the predictive models. PCA with different dimensions had a relatively clear effect on the predictive accuracy. Interestingly, this effect seemed to follow the “elbow rule”, implying that there is a specific dimension value at which the dimensionality-reduced model can achieve the best predictive performance [49,50]. However, the “elbow” positions for different traits were inconsistent and exhibited no clear pattern. On the other hand, surprisingly, both locus filtering based on MAF and LD pruning appeared to have no significant effect on predictive accuracy. Within our study population, MAF was used as a preliminary criterion for judging whether loci were affected by artificial selection, although this was not precise [51,52]. It is generally considered that loci under strong artificial selection would show reduced polymorphism, reflected in lower MAF values [53,54]. We speculate that the reason for this phenomenon might be that the breeding purposes of this population were primarily concentrated on EN traits, with less selection for other traits. Therefore, the MAF values of loci related to these traits were almost uniformly distributed between 0.1 and 0.5 and thus did not have a definitive effect on model accuracy.

The GWAS identified SNPs that were significantly associated with various traits across the entire genome. Applying these suggestive significant SNPs to ML models improved the predictive accuracy and reduced the prediction biases for nearly all the traits, which is consistent with our expectations. In our results, direct genomic prediction using genotypes of these SNPs reaped better performance in most phenotypes compared to using eigenvalues from PCA. In SINS and ESCL, the improvements in predictive accuracy were striking, even approaching three times that of the previous method (188~277%). This result indicates that the GWAS can serve as a feature-engineering method, playing a role in genomic prediction models. However, it is worth noting that these values are not exact values of predictive accuracy for traits but rather the magnitude of improvement compared to previous methods. Considering that typical GWASs use linear mixed models and control for population structure and false-positive correction in SNPs, the identified significant and suggestive significant loci have a clear statistical association with traits. This not only helps ML models to reduce the need to process redundant information but also focuses on feature extraction, thereby obtaining more accurate predictive results.

## 5. Conclusions

Our study revealed that ML algorithms outperformed the traditional rrBLUP and BayesA in predicting economic traits using genotype data in a Rhode Island Red female chicken population. We found that ML performed better at predicting most traits using eigenvalues from PCA. It also outperformed rrBLUP and BayesA in more than half the phenotypes, especially BW and EW. In addition, the application of suggestive significant SNPs obtained from the GWAS to genomic prediction substantially improved the effect of prediction, suggesting that the GWAS could be involved in genomic prediction as a feature extraction method. In conclusion, this study strengthens our understanding of ML algorithms and feature engineering in animal genome prediction and provides valuable insights for future research in this area.

## Figures and Tables

**Table 1:** Hyperparameter table for the AutoGluon and ML models.

Model	Parameter Name	Parameter
AutoGluon	eval_metric	root_mean_squared_error
		pearsonr
	presets	best_quality
	num-bag-folds	10
CatBoost (CAT)	iterations	500
	learning_rate	0.009
	depth	6
	random_strength	1.0
	max_leaves	31
	rsm	1.0
	sampling_frequency	PerTreeLevel
	bagging_temperature	1.0
	grow_policy	SymmetricTree
Extra Tree (ET)	n_estimators	500
	max_depth	None
	min_samples_split	2
	min_samples_leaf	1
	max_features	1
K-Nearest Neighbors (KNNs)	K	50
LightGBM (LGB)	num_boost_round	100
	learning_rate	0.1
	num_leaves	64
	feature_fraction	0.9
	bagging_fraction	0.9
	max_depth	6
	min_data_in_leaf	3
	boosting	gbdt
NNFastAi (NN)	y_scaler	-
	clipping	-
	layers	32
	emb_drop	0.1
	ps	0.1
	bs	256
	epochs	150
Random Forest (RF)	n_estimators	500
	min_samples_split	2
	min_samples_leaf	1
	max_features	1
	max_depth	None

**Table 2:** Descriptive statistics of important economic traits of chickens along with the aging process.

Traits	N	Mean	SD	CV (%)	Min	Max	h^2^
AH36	2178	6.87	1.27	18.54%	2.1	10.8	0.187
AH56	2245	7.10	1.05	14.77%	2.2	11	0.266
AH72	2367	6.02	1.29	21.37%	2.2	11.1	0.258
AH80	2207	5.85	1.47	25.17%	1	13.5	0.157
BW28	4186	1968	147.02	7.47%	1125	2649	0.442
BW36	4189	2043	171.40	8.39%	1575	2638	0.524
BW56	4014	2146	222.51	10.37%	1299	3119	0.328
BW72	3282	2178	224.35	10.30%	1271	2874	0.323
BW80	3705	2215	228.53	10.32%	1033	3088	0.387
BWAFE	4189	1782	113.12	6.35%	1030	2265	0.446
EN38	4190	123.3	7.70	6.24%	100	146	0.436
EN48	4190	188.2	9.51	5.06%	131	217	0.409
EN56	4190	238.1	12.97	5.45%	146	270	0.336
EN72	3833	339	22.63	6.67%	190	379	0.142
ESCA36	2469	17.42	1.35	7.76%	12.61	21.5	0.437
ESCA56	1445	16.94	1.72	10.17%	2.17	23.5	0.464
ESCA72	2493	17.02	2.05	12.04%	2.51	22.18	0.315
ESCA80	2886	16.54	1.89	11.44%	5.29	21.79	0.360
ESCB36	2469	28.79	1.40	4.85%	19.99	32.81	0.446
ESCB56	1445	28.68	1.69	5.88%	12.39	32.75	0.399
ESCB72	2493	28.74	1.97	6.86%	10.01	33.83	0.242
ESCB80	2886	28.12	1.86	6.63%	15.01	32.19	0.254
ESCI36	2469	12.86	4.39	34.13%	−0.43	28.46	0.435
ESCI56	1445	16.71	5.51	32.95%	1.75	63.83	0.349
ESCI72	2510	15.47	6.87	44.44%	1.24	70.19	0.227
ESCI80	2886	16.05	6.47	40.32%	−0.57	55.31	0.295
ESCL36	4149	59.15	3.02	5.11%	47.39	73	0.364
ESCL56	2614	62.42	3.43	5.50%	50.97	78.7	0.455
ESCL72	2992	61.72	3.67	5.95%	51.57	82.71	0.496
ESCL80	3336	60.87	3.83	6.29%	49.13	85.12	0.414
ESS36	3130	3.151	0.70	22.14%	1.049	5.401	0.317
ESS56	2858	3.203	0.74	23.04%	0.89	5.456	0.669
ESS72	2873	3.017	0.75	24.91%	0.803	5.224	0.552
ESS80	3304	2.752	0.71	25.69%	0.515	5.182	0.698
EW28	4158	56.97	3.51	6.16%	40	89.7	0.436
EW36	4172	58.41	3.77	6.45%	40	75	0.448
EW56	3905	60.44	4.47	7.39%	35	80.6	0.581
EW72	2855	61.51	4.69	7.62%	35	84.7	0.440
EW80	3155	61.86	4.75	7.68%	35	81.5	0.455
EWAFE	4173	43.06	5.88	13.65%	18.9	88	0.178
SINS36	856	3.167	0.59	18.60%	1.87	5.6	0.272
SINS56	685	2.45	0.48	19.69%	1.4	4.8	0.290
SINS72	1631	3.545	1.47	41.34%	1.3	11	0.301
SINS80	2037	2.494	0.57	22.84%	1.15	4.93	0.410

N, number of samples. Mean, average value. SD, standard deviation. CV (%), coefficient of variation. Min, minimum value. Max, maximum value. h^2^, heritability. AH, albumen height (mm); BW, body weight (g); AFE, age at first egg; EN, egg number; ESC: eggshell color; ESS, eggshell strength; EW, egg weight (g); HU, Haugh unit; SINS, eggshell gloss. The number following the trait indicates the age of week.

**Table 3:** Functional annotation of genes associated with suggestive significant SNPs from the GWAS.

Traits	Source	Term Name	Term ID	*p* Value
AH	GO:MF	molecular function	GO:0003674	9.62 × 10^−13^
	GO:MF	heparin binding	GO:0008201	1.42 × 10^−2^
	GO:BP	biological process	GO:0008150	6.33 × 10^−11^
	GO:CC	cellular anatomical entity	GO:0110165	9.98 × 10^−15^
BW	GO:MF	molecular function	GO:0003674	7.48 × 10^−29^
	GO:MF	RNA polymerase II transcription regulatory region sequence-specific DNA binding	GO:0000977	1.15 × 10^−2^
	GO:BP	biological process	GO:0008150	1.09 × 10^−20^
	GO:BP	cell–cell junction organization	GO:0045216	5.50 × 10^−5^
	GO:BP	inorganic cation transmembrane transport	GO:0098662	5.78 × 10^−3^
	GO:BP	regulation of receptor-mediated endocytosis	GO:0048259	4.96 × 10^−2^
	GO:CC	cellular component	GO:0005575	3.08 × 10^−22^
EN	GO:MF	molecular function	GO:0003674	9.64 × 10^−9^
	GO:BP	cellular process	GO:0009987	8.63 × 10^−10^
	GO:BP	cellular response to salt	GO:1902075	3.00 × 10^−2^
	GO:CC	cellular anatomical entity	GO:0110165	2.66 × 10^−8^
	GO:CC	MOZ/MORF histone acetyltransferase complex	GO:0070776	1.12 × 10^−2^
ESC	GO:MF	molecular function	GO:0003674	2.24 × 10^−64^
	GO:MF	monoatomic cation channel activity	GO:0005261	8.11 × 10^−3^
	GO:MF	adenylate cyclase regulator activity	GO:0010854	1.17 × 10^−2^
	GO:MF	tubulin binding	GO:0015631	2.41 × 10^−2^
	GO:MF	metal ion transmembrane transporter activity	GO:0046873	2.80 × 10^−2^
	GO:BP	biological process	GO:0008150	3.04 × 10^−58^
	GO:BP	inorganic ion homeostasis	GO:0098771	2.09 × 10^−3^
	GO:BP	negative regulation of cytoskeleton organization	GO:0051494	8.00 × 10^−3^
	GO:CC	cellular anatomical entity	GO:0110165	4.66 × 10^−47^
ESS	GO:MF	binding	GO:0005488	1.94 × 10^−11^
	GO:MF	voltage-gated calcium channel activity involved in cardiac muscle cell action potential	GO:0086007	2.98 × 10^−5^
	GO:MF	sequence-specific double-stranded DNA binding	GO:1990837	2.75 × 10^−3^
	GO:MF	histone reader activity	GO:0140566	2.76 × 10^−2^
	GO:BP	biological process	GO:0008150	1.11 × 10^−12^
	GO:BP	myeloid cell differentiation	GO:0030099	1.23 × 10^−3^
	GO:BP	membrane depolarization during cardiac muscle cell action potential	GO:0086012	2.49 × 10^−3^
	GO:BP	cell–cell signaling involved in cardiac conduction	GO:0086019	1.98 × 10^−2^
	GO:CC	cellular anatomical entity	GO:0110165	8.57 × 10^−17^
	GO:CC	clathrin-coated pit	GO:0005905	3.80 × 10^−2^
	GO:CC	monoatomic ion channel complex	GO:0034702	4.13 × 10^−2^
EW	GO:MF	binding	GO:0005488	2.73 × 10^−30^
	GO:MF	transcription coactivator activity	GO:0003713	1.68 × 10^−4^
	GO:MF	frizzled binding	GO:0005109	1.94 × 10^−2^
	GO:BP	biological process	GO:0008150	9.56 × 10^−31^
	GO:BP	response to oxygen-containing compound	GO:1901700	5.21 × 10^−3^
	GO:BP	peptidyl–threonine modification	GO:0018210	4.36 × 10^−2^
	GO:BP	cellular response to insulin stimulus	GO:0032869	4.43 × 10^−2^
	GO:CC	cellular anatomical entity	GO:0110165	1.23 × 10^−38^
SINS	GO:MF	molecular function	GO:0003674	1.19 × 10^−16^
	GO:MF	dipeptidyl–peptidase activity	GO:0008239	1.86 × 10^−2^
	GO:BP	biological process	GO:0008150	2.87 × 10^−15^
	GO:BP	intracellular signal transduction	GO:0035556	8.65 × 10^−3^
	GO:BP	gene expression	GO:0010467	1.18 × 10^−2^
	GO:CC	cellular component	GO:0005575	6.02 × 10^−12^
	GO:CC	lamellipodium membrane	GO:0031258	3.73 × 10^−2^

**Table 4:** Predictive accuracy and biases of all candidate traits.

Traits	CAT	ET	KNN	LGB	NN	RF	WE	rrBLUP	BayesA
AH36	0.32 (1.21)	0.24 (1.24)	0.25 (1.31)	0.17 (1.21)	0.17 (1.27)	0.32 (1.21)	0.33 (1.21)	0.23 (0.73)	0.22 (0.76)
AH56	0.3 (0.87)	0.35 (0.86)	0.16 (0.98)	0.24 (0.87)	0.38 (0.85)	0.32 (0.87)	0.35 (0.86)	0.32 (0.79)	0.32 (0.8)
AH72	0.09 (1.29)	0.06 (1.29)	−0.02 (1.46)	0.03 (1.29)	0.09 (1.28)	0.19 (1.27)	0.07 (1.28)	0.22 (0.67)	0.22 (0.69)
AH80	0.30 (1.39)	0.38 (1.36)	0.24 (1.49)	0.2 (1.38)	0.24 (1.42)	0.37 (1.35)	0.38 (1.36)	0.26 (0.65)	0.25 (0.69)
BW28	0.32 (138.03)	0.24 (141.35)	0.27 (147.12)	0.27 (141.21)	0.24 (141.33)	0.26 (140.55)	0.33 (137.41)	0.25 (201.87)	0.27 (203.69)
BW36	0.42 (151.99)	0.45 (149.78)	0.36 (164.36)	0.45 (149.95)	0.32 (156.9)	0.44 (150.77)	0.44 (152.23)	0.3 (224.72)	0.27 (238.91)
BW56	0.25 (216.9)	0.19 (220.08)	0.19 (233.42)	0.22 (218.92)	0.17 (224.95)	0.22 (218.65)	0.26 (216.58)	0.16 (228.27)	0.27 (236.08)
BW72	0.16 (221.8)	0.18 (221.41)	0.07 (246.96)	0.17 (221.5)	0.17 (221.56)	0.22 (219.31)	0.18 (220.76)	0.14 (236.28)	0.28 (252.72)
BW80	0.18 (210.63)	0.15 (211.38)	0.28 (217.12)	0.18 (213.07)	0.16 (211.72)	0.16 (211.3)	0.27 (205.85)	0.15 (237.68)	0.22 (247.77)
BWAFE	0.26 (109.82)	0.29 (109.71)	0.28 (112.45)	0.24 (109.34)	0.24 (110.89)	0.33 (108.35)	0.35 (107.11)	0.34 (180.89)	0.34 (180.55)
EN38	0.04 (8.15)	0.05 (8.43)	0.19 (9.09)	0.12 (8.39)	0 (7.8)	0.17 (7.98)	0.12 (7.98)	0.27 (12.31)	0.3 (12.51)
EN48	0 (10.32)	0.09 (10.32)	0.2 (10.92)	0.05 (10.56)	−0.01 (9.86)	0.09 (10.31)	0.17 (9.96)	0.25 (18.8)	0.28 (18.99)
EN56	0.03 (12.64)	0.07 (12.89)	0.16 (14.77)	0.15 (13.53)	0.04 (12.22)	0.09 (13.18)	0.11 (12.28)	0.19 (23.75)	0.21 (23.99)
EN72	0.05 (23.63)	0 (24.64)	0.07 (28.15)	0 (24.59)	−0.01 (22.95)	0.06 (23.55)	0.06 (22.96)	0.16 (33.51)	0.15 (33.57)
ESCA36	0.35 (1.19)	0.31 (1.23)	0.38 (1.2)	0.36 (1.18)	0.24 (1.24)	0.39 (1.17)	0.42 (1.17)	0.24 (1.76)	0.22 (1.77)
ESCA56	0.29 (1.59)	0.3 (1.58)	0.27 (1.67)	0.33 (1.59)	0.05 (1.66)	0.28 (1.61)	0.24 (1.62)	0.24 (1.74)	0.24 (1.74)
ESCA72	0.28 (1.88)	0.29 (1.88)	0.29 (1.97)	0.21 (1.88)	0.22 (1.96)	0.3 (1.87)	0.31 (1.87)	0.36 (1.76)	0.36 (1.76)
ESCA80	0.3 (1.75)	0.36 (1.72)	0.19 (1.91)	0.33 (1.73)	0.3 (1.76)	0.33 (1.74)	0.32 (1.75)	0.37 (1.72)	0.36 (1.73)
ESCB36	0.32 (1.24)	0.34 (1.23)	0.33 (1.3)	0.29 (1.25)	0.24 (1.27)	0.33 (1.24)	0.35 (1.23)	0.24 (2.91)	0.24 (2.92)
ESCB56	0.24 (1.51)	0.11 (1.56)	0.26 (1.56)	0.08 (1.53)	0.07 (1.56)	0.21 (1.56)	0.2 (1.52)	0.19 (2.89)	0.17 (2.9)
ESCB72	0.03 (1.85)	0.13 (1.85)	0.19 (1.94)	0.04 (1.85)	0.06 (1.94)	0.11 (1.87)	0.1 (1.88)	0.22 (2.9)	0.22 (2.9)
ESCB80	0.11 (1.85)	0.18 (1.83)	0.07 (1.98)	0.02 (1.86)	0.15 (1.87)	0.09 (1.87)	0.14 (1.85)	0.23 (2.87)	0.23 (2.89)
ESCI36	0.27 (3.91)	0.26 (3.95)	0.29 (4.03)	0.08 (3.91)	0.22 (3.91)	0.32 (3.89)	0.33 (3.85)	0.26 (1.27)	0.25 (1.27)
ESCI56	0.1 (5.45)	−0.09 (5.64)	0.11 (5.81)	0.23 (5.45)	0.01 (5.73)	0 (5.59)	0.11 (5.64)	0.2 (1.67)	0.19 (1.66)
ESCI72	0.26 (6.4)	0.28 (6.37)	0.26 (6.73)	0.24 (6.35)	0.14 (6.68)	0.23 (6.51)	0.24 (6.42)	0.32 (1.56)	0.33 (1.69)
ESCI80	0.34 (5.75)	0.4 (5.67)	0.16 (6.41)	0.34 (5.76)	0.3 (5.82)	0.37 (5.7)	0.38 (5.73)	0.37 (1.53)	0.36 (1.59)
ESCL36	0.37 (2.84)	0.27 (2.93)	0.26 (3.07)	0.14 (2.85)	0.24 (2.93)	0.35 (2.86)	0.33 (2.86)	0.38 (5.89)	0.37 (5.91)
ESCL56	0.2 (3.46)	0.23 (3.45)	0.22 (3.6)	0.19 (3.48)	0.22 (3.51)	0.19 (3.47)	0.26 (3.4)	0.36 (6.23)	0.35 (6.28)
ESCL72	0.34 (3.59)	0.33 (3.58)	0.23 (3.86)	0.34 (3.58)	0.3 (3.64)	0.33 (3.58)	0.36 (3.55)	0.44 (6.06)	0.45 (6.13)
ESCL80	0.34 (3.69)	0.38 (3.66)	0.26 (3.98)	0.27 (3.71)	0.21 (3.88)	0.34 (3.69)	0.36 (3.66)	0.42 (6.02)	0.43 (6.08)
ESS36	0.52 (0.56)	0.54 (0.55)	0.48 (0.59)	0.42 (0.56)	0.39 (0.6)	0.52 (0.56)	0.54 (0.55)	0.39 (0.33)	0.39 (0.33)
ESS56	0.42 (0.7)	0.43 (0.69)	0.39 (0.72)	0.37 (0.69)	0.24 (0.77)	0.43 (0.69)	0.44 (0.69)	0.22 (0.32)	0.23 (0.31)
ESS72	0.41 (0.69)	0.49 (0.66)	0.4 (0.71)	0.39 (0.67)	0.28 (0.73)	0.48 (0.67)	0.44 (0.68)	0.29 (0.3)	0.27 (0.29)
ESS80	0.49 (0.67)	0.51 (0.65)	0.45 (0.68)	0.44 (0.65)	0.2 (0.76)	0.5 (0.66)	0.51 (0.65)	0.24 (0.29)	0.24 (0.28)
EW28	0.3 (3.3)	0.26 (3.32)	0.19 (3.57)	0.15 (3.3)	0.21 (3.39)	0.19 (3.43)	0.29 (3.3)	0.33 (5.76)	0.34 (5.76)
EW36	0.3 (3.84)	0.32 (3.84)	0.25 (4.07)	0.31 (3.83)	0.3 (3.95)	0.3 (3.85)	0.34 (3.82)	0.33 (5.91)	0.33 (5.93)
EW56	0.37 (4.31)	0.37 (4.3)	0.31 (4.57)	0.35 (4.34)	0.24 (4.48)	0.36 (4.32)	0.38 (4.3)	0.36 (6.1)	0.36 (6.12)
EW72	0.24 (4.53)	0.29 (4.47)	0.33 (4.61)	0.27 (4.46)	0.29 (4.47)	0.25 (4.51)	0.35 (4.39)	0.25 (6.14)	0.25 (6.16)
EW80	0.23 (4.56)	0.28 (4.49)	0.27 (4.79)	0.31 (4.42)	0.19 (4.64)	0.29 (4.48)	0.26 (4.51)	0.27 (6.19)	0.27 (6.21)
EWAFE	0.09 (6.36)	0.01 (6.43)	0.04 (6.9)	0.08 (6.34)	−0.01 (6.42)	−0.01 (6.56)	0.02 (6.38)	0.17 (4.3)	0.17 (4.25)
SINS36	0.06 (0.53)	−0.03 (0.55)	−0.14 (0.63)	0.09 (0.53)	0.03 (0.54)	0.02 (0.55)	−0.04 (0.55)	0.05 (0.32)	0.02 (0.33)
SINS56	−0.03 (0.57)	−0.14 (0.57)	0.1 (0.59)	−0.02 (0.56)	0.05 (0.57)	−0.09 (0.58)	0.04 (0.56)	0.06 (0.25)	0.05 (0.25)
SINS72	0.71 (0.96)	0.71 (0.96)	0.65 (1.07)	0.69 (0.98)	0.62 (1.06)	0.72 (0.95)	0.71 (0.95)	0.49 (0.36)	0.61 (0.53)
SINS80	0.16 (0.59)	0.21 (0.59)	0.29 (0.61)	0.22 (0.58)	0.25 (0.59)	0.25 (0.58)	0.29 (0.58)	0.33 (0.27)	0.32 (0.28)

Numbers outside the parentheses are the Pearson correlation coefficient, and the ones on the inside are the RMSE.

## Data Availability

The datasets generated and/or analyzed during the current study are not publicly available due to commercial confidentiality but are available from the corresponding author upon reasonable request.

## Supplementary Materials

The following supporting information can be downloaded at https://www.mdpi.com/article/10.3390/genes15060690/s1. Figure S1: Predictive accuracy of ML models for 56-week traits using all SNPs; Figure S2: Prediction biases of ML models for 56-week traits using all SNPs; Figure S3: Predictive accuracy of the WE model for traits at 56 weeks using different PCA methods; Figure S4: Predictive accuracy of the WE model for traits at 56 weeks using different MAF and LD pruning methods; Table S1: Significant and suggestive significant SNPs identified by GWAS for all phenotypes; Table S2: Genes in which significant loci were located.
