# Supplementary figures and images for: Integrating Bioinformatics and Machine Learning for Genomic Prediction in Chickens
Source: Genes (Basel). 2024 May 26;15(6):690. doi: 10.3390/genes15060690 (PMC11202573; doi:10.3390/genes15060690)

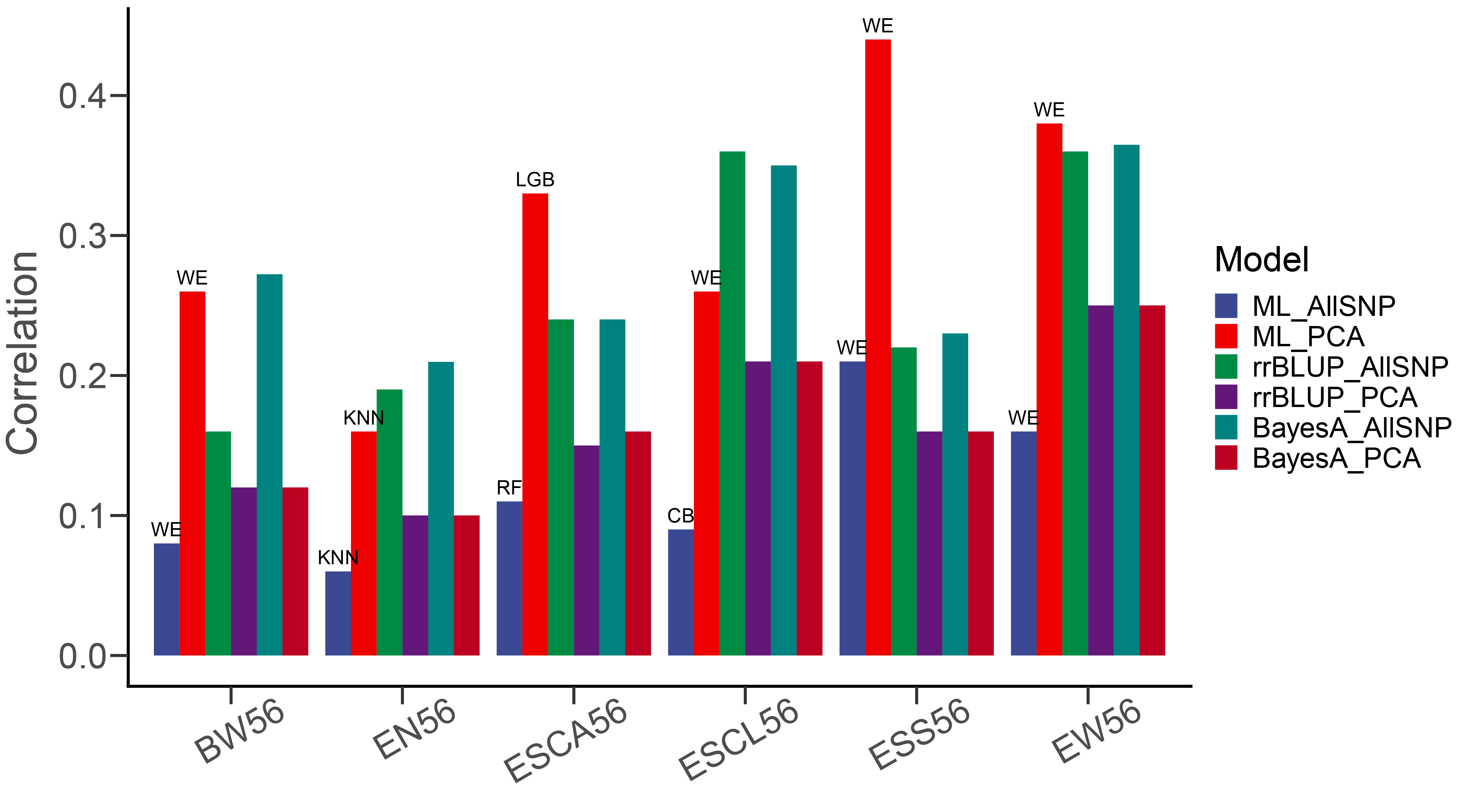

Supplement: Supplementary file 1 [file genes-15-00690-s001.zip › FigS1.png]

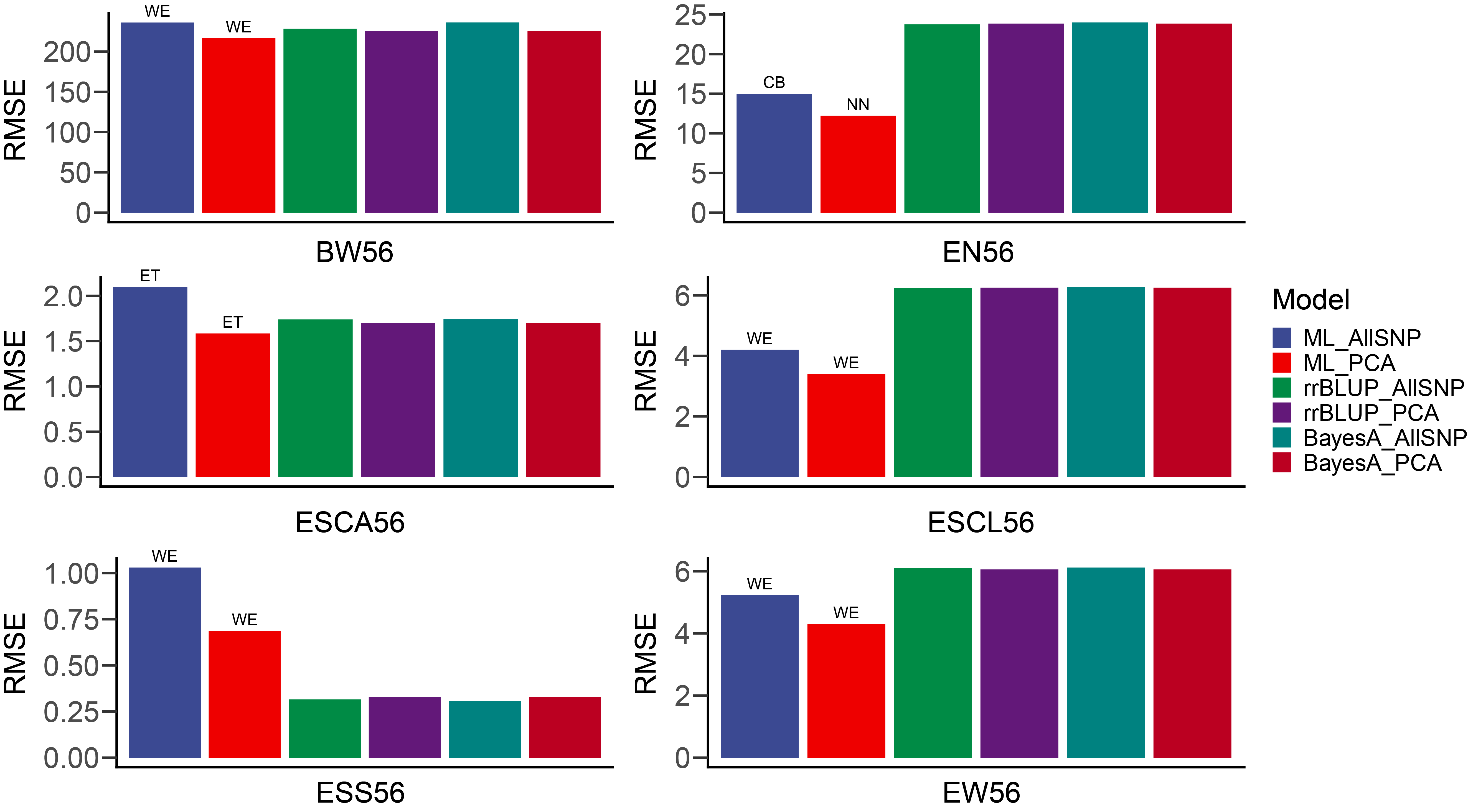

Supplement: Supplementary file 1 [file genes-15-00690-s001.zip › FigS2.png]

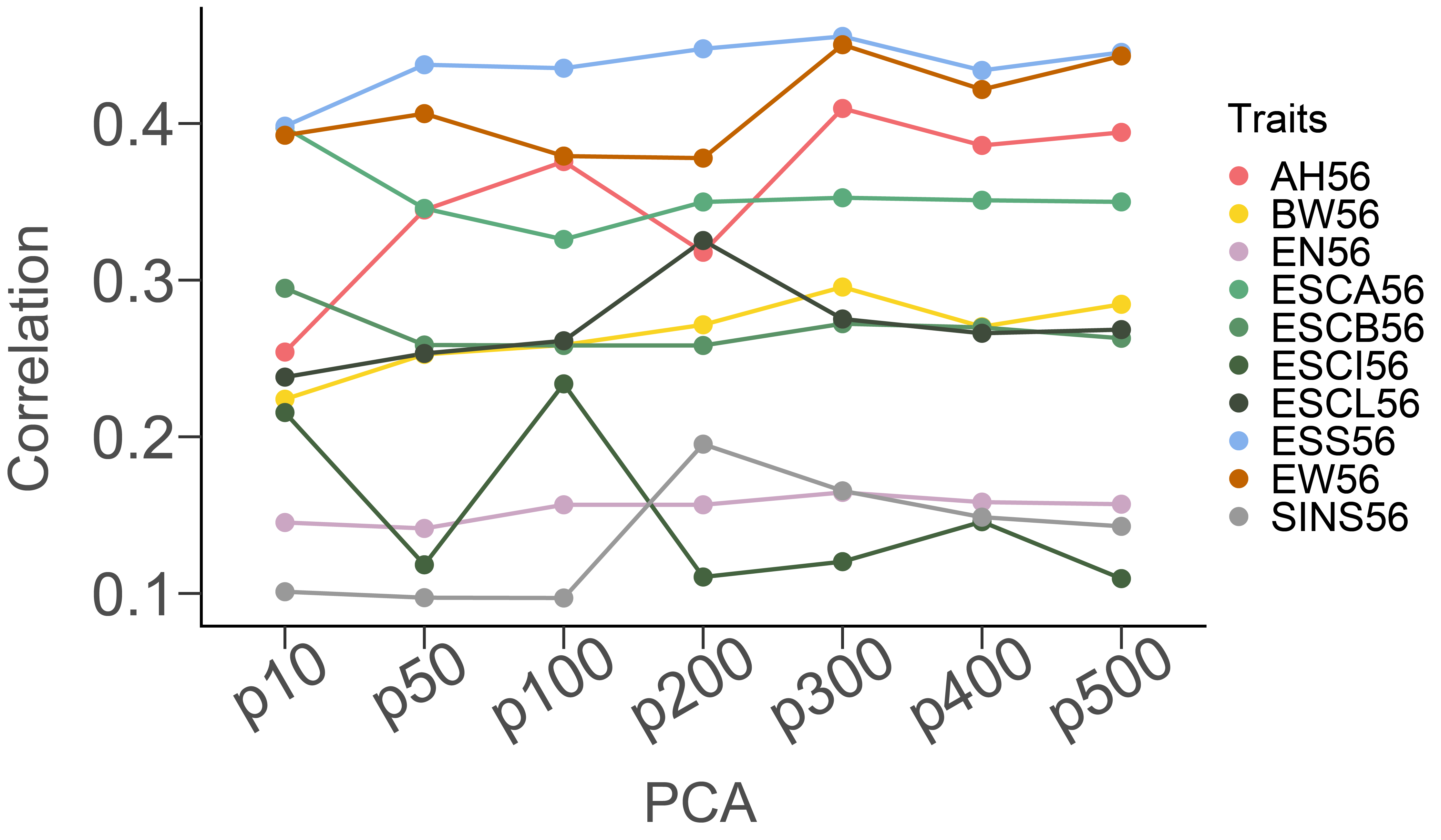

Supplement: Supplementary file 1 [file genes-15-00690-s001.zip › FigS3.png]

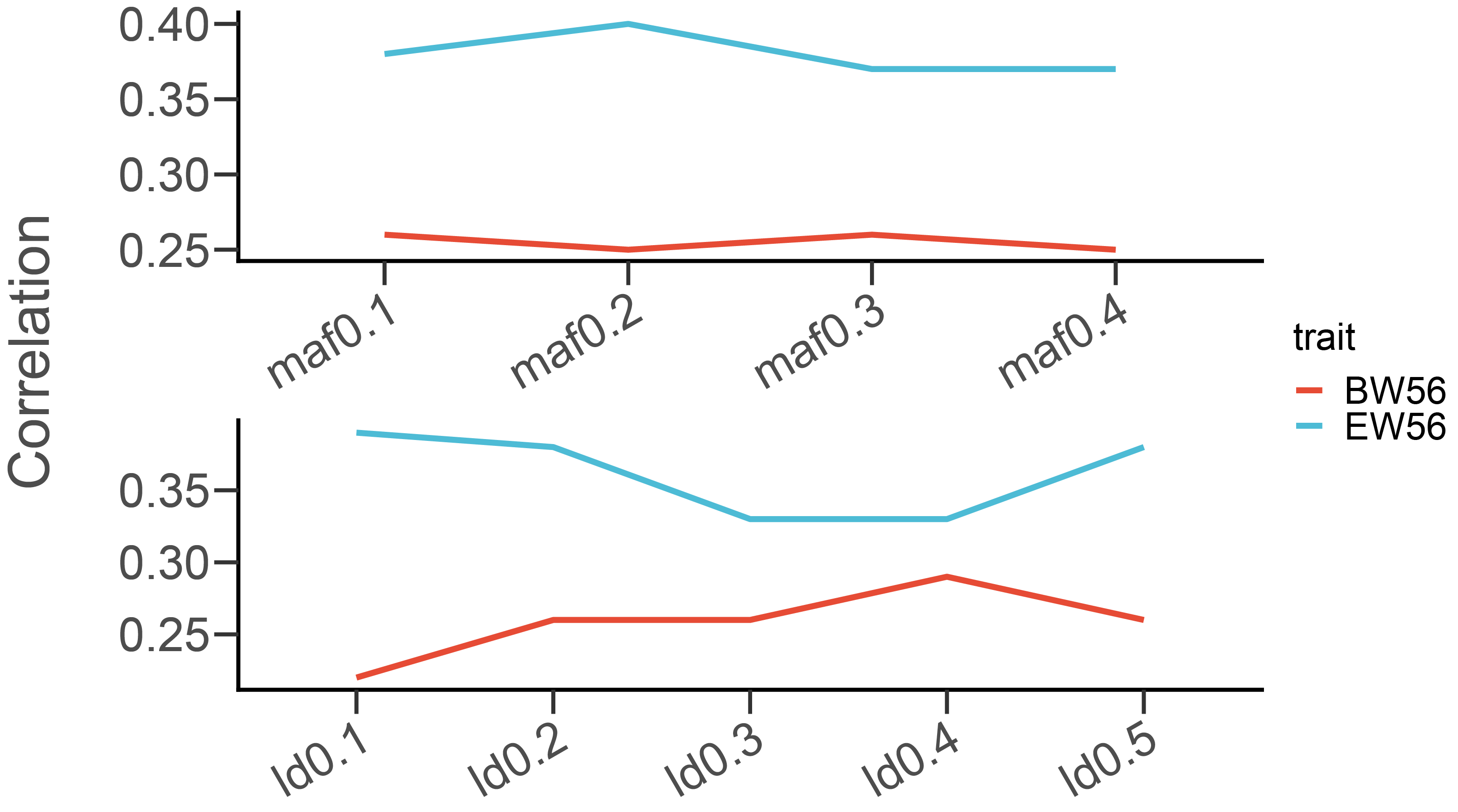

Supplement: Supplementary file 1 [file genes-15-00690-s001.zip › FigS4.png]
